# Molecular dynamics simulation study on the effect of perfluorosulfonic acid side chains on oxygen permeation in hydrated ionomers of PEMFCs

**DOI:** 10.1038/s41598-021-87570-8

**Published:** 2021-04-22

**Authors:** Sung Hyun Kwon, Haisu Kang, Young-Jun Sohn, Jinhee Lee, Sunbo Shim, Seung Geol Lee

**Affiliations:** 1 School of Chemical Engineering, Pusan National University, 2, Busandaehak-ro 63beon-gil, Geumjeong-gu, Busan, 46241 Republic of Korea; 2 Fuel Cell Laboratory, Korea Institute of Energy Research (KIER), Yuseong-gu, Daejeon, 34129 Republic of Korea; 3 Advanced Energy and System Engineering, University of Science and Technology, Gajeong-ro 217, Yuseong-gu, Daejeon, 34113 Daejeon, Republic of Korea; 4 Hyundai Motor Company, 17-5, Mabuk-ro 240, Giheung-gu, Yongin-si, Gyeonggi-do, Yongin-si, 16891 Republic of Korea; 5 Department of Organic Material Science and Engineering, Pusan National University, 2, Busandaehak-ro 63beon-gil, Geumjeong-gu, Busan, 46241 Republic of Korea

**Keywords:** Soft materials, Theory and computation

## Abstract

We prepared two types of perfluorosulfonic acid (PFSA) ionomers with Aquivion (short side chain) and Nafion (long side chain) on a Pt surface and varied their water contents (2.92 ≤ *λ* ≤ 13.83) to calculate the solubility and permeability of O_2_ in hydrated PFSA ionomers on a Pt surface using full atomistic molecular dynamics (MD) simulations. The solubility and permeability of O_2_ molecules in hydrated Nafion ionomers were greater than those of O_2_ molecules in hydrated Aquivion ionomers at the same water content, indicating that the permeation of O_2_ molecules in the ionomers is affected not only by the diffusion coefficient of O_2_ but also by the solubility of O_2_. Notably, O_2_ molecules are more densely distributed in regions where water and hydronium ions have a lower density in hydrated Pt/PFSA ionomers. Radial distribution function (RDF) analysis was performed to investigate where O_2_ molecules preferentially dissolve in PFSA ionomers on a Pt surface. The results showed that O_2_ molecules preferentially dissolved between hydrophilic and hydrophobic regions in a hydrated ionomer. The RDF analysis was performed to provide details of the O_2_ location in hydrated PFSA ionomers on a Pt surface to evaluate the influence of O_2_ solubility in ionomers with side chains of different lengths. The coordination number of C(center)–O(O_2_) and O(side chain)–O(O_2_) pairs in hydrated Nafion ionomers was higher than that of the same pairs in hydrated Aquivion ionomers with the same water content. Our investigation provides detailed information about the properties of O_2_ molecules in different PFSA ionomers on a Pt surface and with various water contents, potentially enabling the design of better-performing PFSA ionomers for use in polymer electrolyte membrane fuel cells.

## Introduction

Polymer electrolyte membrane fuel cells (PEMFCs) are environmentally friendly energy sources that can alleviate environmental problems because of their low emissions of environmental pollutant gases such as SO_*x*_, NO_*x*_, CO_2_, and CO^1,2^. PEMFCs have been used in various applications such as fuel-cell vehicles and power supplies (including portable power supplies) because, in addition to their ecofriendly benefits, PEMFCs can also generate high power densities and operate with short start-up times because of their low operating temperature^1,3–5^. In general, PEMFCs consist of membrane electrode assembly (MEA) layers, gas-diffusion layers, microporous layers, gas flow channels, and bipolar plates^3^. The MEA layers are especially important because the cell performance and durability of PEMFCs are strongly affected by the design and composition of their MEA layers^4^. MEA layers in a PEMFC system comprise catalyst layers (CLs) and a polymer membrane; the polymer membrane plays a critical role in transferring protons from the anode to the cathode in the process of generating electricity^5^. CLs also play a critical role because the electrochemical reactions related to energy conversion in a PEMFC, such as the hydrogen oxidation reaction or oxygen reduction reaction (ORR), occur in CLs. The structure of CLs includes a carbon matrix with a Pt catalyst (Pt/C) and proton-conducting ionomers. Importantly, the proton transfer performance of a CL is affected by its ionomer thin film on Pt/C because protons can directly reach the Pt surface through the hydrated ionomer thin film. Therefore, the composition and morphology of the ionomers strongly influence the performance of a PEMFC.

The proton-conducting ionomers in PEMFCs are categorized as a perfluorinated acid (PFSA), nonfluorinated hydrocarbon, or an acid–base complex^1^. Among these various polymer ionomers, PFSA ionomers such as Nafion (DuPont)^6,7^, Aquivion (Dow Chemical)^8,9^, Aciplex-S (Asahi Glass)^10^, and Flemion (Asahi Glass)^10,11^ have been extensively used in PEMFCs because of their excellent proton conductivity and good mechanical, chemical, and thermal stabilities^12^. PFSA-based ionomers are composed of main chains of polytetrafluoroethylene (PTFE) and side chains terminated by sulfonic acid groups. Both experimental^13–15^ and theoretical^16–24^ investigations have been performed to elucidate the proton transfer and O_2_ permeation mechanisms in PEMFC systems because the length of the side chains in PFSA ionomers affects PEMFC performance.

Garsany et al.^13^ and Siracusano et al.^14^ experimentally investigated PFSA ionomers—specifically, Aquivion, which has a short side chain, and Nafion, which has a long side chain—to improve PEMFC performance. They concluded that Aquivion exhibits better cell performance than Nafion in PEMFCs because the Aquivion ionomer in the cathode CLs has a lower proton transport resistance, lower charge transfer resistance for the ORR, and lower mass transport resistance than the Nafion ionomer. Baschetti et al.^15^ investigated gas permeation in Nafion and Aquivion ionomers at various temperatures and relative humidities. Humidity and temperature have especially strong effects on gas permeability, and Nafion 117 ionomer was found to exhibit greater O_2_ gas permeability than Aquivion at 50 ℃.

Several groups have investigated the relationship between the diffusion coefficients of water and hydronium ions and PFSA morphologies in systems with various water contents and at different temperatures using molecular dynamics (MD) simulations^16–21^. The diffusion coefficients of water and hydronium ions were found to increase with increasing PFSA water content and increasing temperature. In addition, the sulfur–sulfur interatomic distance in PFSA increased with increasing PFSA water content. MD simulations^22–24^ have also been performed to investigate O_2_ permeation in Nafion ionomers in CLs. Kurihara et al.^22,23^ investigated the permeation of O_2_ gas into a Nafion ionomer on a Pt surface using MD simulations because such simulations are useful for understanding the nanoscale structures in the CLs of PEMFCs. They concluded that the diffusion coefficient of O_2_ molecules increased and the solubility of O_2_ molecules decreased with increasing water content in the Nafion ionomer. Jinnouchi et al.^24^ also used MD simulations to investigate O_2_ permeation through a Nafion thin film on a Pt surface, where the water content of the Nafion film was varied. Their results indicated that O_2_ permeation in Nafion increased with increasing water content and that understanding the behavior of O_2_ in PFSA ionomers on a Pt surface is critical to understanding its permeation properties. Therefore, the aforementioned experimental results indicate that the length of the side chain in PFSA ionomers can affect both the performance of PEMFCs and the O_2_ permeation behavior. Therefore, studies comparing of the O_2_ permeation properties of Nafion and Aquivion are needed to elucidate the effect of side-chain length in PFSA ionomers in PEMFCs.

In the present study, computational simulations using the full atomistic MD simulation technique are carried out to obtain detailed molecular information for calculating the transport properties of hydrated PFSA ionomers with various water contents on a Pt surface. In addition, the O_2_ permeation properties of hydrated PFSA with different side-chain lengths were measured at the interfacial region on the Pt surface. Therefore, two types of PFSA ionomers—Nafion (longer side chain) and Aquivion (shorter side chain)—were prepared for measurement of the O_2_ permeability at various water contents, enabling the relationship between the hydrated PFSA structure and the O_2_ permeation properties to be elucidated. In addition, the distribution of O_2_ and water in PFSA ionomers on a Pt surface were also analyzed using density profiles and radial distribution functions (RDFs) with various water contents at the operating temperature of a PEMFC (353.15 K). We expect that the results of this study will provide detailed information about O_2_ permeability of water-containing PFSA ionomers on a Pt surface and can provide guidance for the design of PFSA ionomers for use in PEMFCs.

## Computational details

### Model preparation

**Figure 1:**
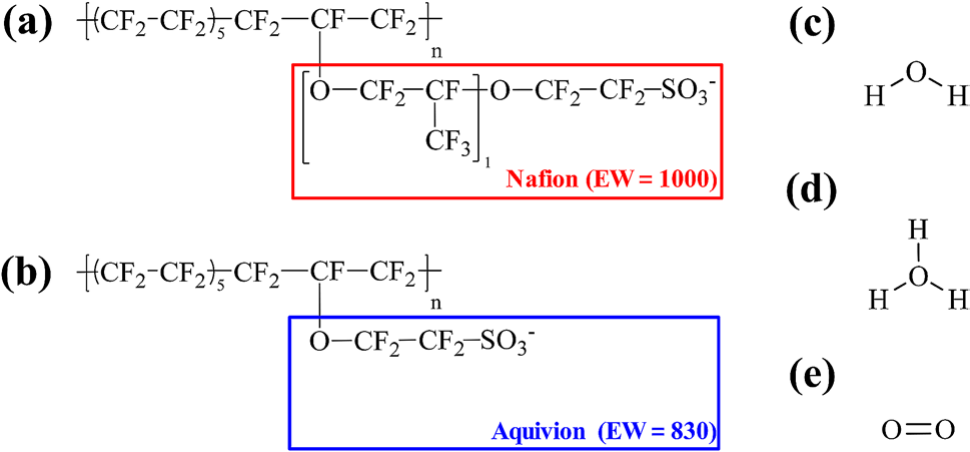
Chemical structures of PFSA component at (**a**) ionized Nafion (EW = 1000), (**b**) ionized Aquivion (EW = 830), and (**c**) water, (**d**) hydronium ion, and (**e**) O_2_ molecules.

Figure 1 shows the chemical structures of the Nafion and Aquivion ionomers. Each ionomer was composed such that each polymer had 10 repeat units with 10 sulfonic acid groups per polymer chain. The molecular weight of the Nafion and Aquivion polymers was 9969.83 g/mol and 8309.63 g/mol per polymer chain; equivalent weights (EWs) of ~ 1000 g/mol and ~ 830 g/mol were applied, respectively. Water, O_2_ molecules, and hydronium ions were prepared for constructing hydrated PFSA ionomers. The components of each PEMFC system are summarized in Table 1. Materials Studio visualization software was used for the atomic visualization^25^.

**Table 1:** Details of parameters for the MD simulations.

	*λ* = 2.92	*λ* = 6.15	*λ* = 9.77	*λ* = 13.83
No. of water molecules	115	309	526	770
No. of hydronium ions	60	60	60	60
No. of O_2_ molecules	735			
No. of PFSA chains (DP 10) (Nafion or Aquivion)	6			

### Force-field and MD parameters

To describe inter- and intramolecular interactions in the Nafion and Aquivion in PEMFC systems, we applied a modified DREIDING force field^26^ in our simulations. The DREIDING force field has been widely used to describe PEMFCs systems^27–33^. The force fields of water molecules and Pt atoms were applied using F3C force field^34^ and the embedded-atom method (EAM) force field, respectively^35^. The total potential energy *E*_total_ in PEMFC systems can be calculated using to Eq. (1):

1 $$E_{{{\text{total}}}} = E_{{{\text{vdW}}}} + E_{{\text{Q}}} + E_{{{\text{bond}}}} + E_{{{\text{angle}}}} + E_{{{\text{torsion}}}} + E_{{{\text{inversion}}}} + E_{{{\text{EAM}}}}$$

where ${E}_{\mathrm{vdW}}$, ${E}_{\mathrm{Q}}$, ${E}_{\mathrm{bond}}$, ${E}_{\mathrm{angle}}$, ${E}_{\mathrm{torsion}}$, ${E}_{\mathrm{inversion}}$, and ${E}_{\mathrm{EAM}}$ are the van der Waals, electrostatic, bond-stretching, angle-bending, torsion, inversion, and the EAM energies, respectively. For calculating entire MD simulations for PEMFC systems, the large-scale atomic/molecular massively parallel simulator (LAMMPS) code^36,37^ from Plimpton at Sandia was used. All MD simulations were carried out using the velocity Verlet algorithm^38^ to integrate equations of atomic motion, with a time steps of 1 fs. The electrostatic interactions in our systems were calculated using the particle–particle particle–mesh method^39^. The charges of particles in Nafion and Aquivion were calculated via density functional theory (DFT) calculations using the Mulliken charge analysis method^40^ in the Materials Studio software^25^. All DFT calculations for charge analyses were carried out using the double numerical basis set with polarization (DNP) function and the generalized gradient approximation with the Perdew–Burke–Ernzerhof functional^41^.

### Force-field parameters between PEMFC components and the Pt surface

**Figure 2:**
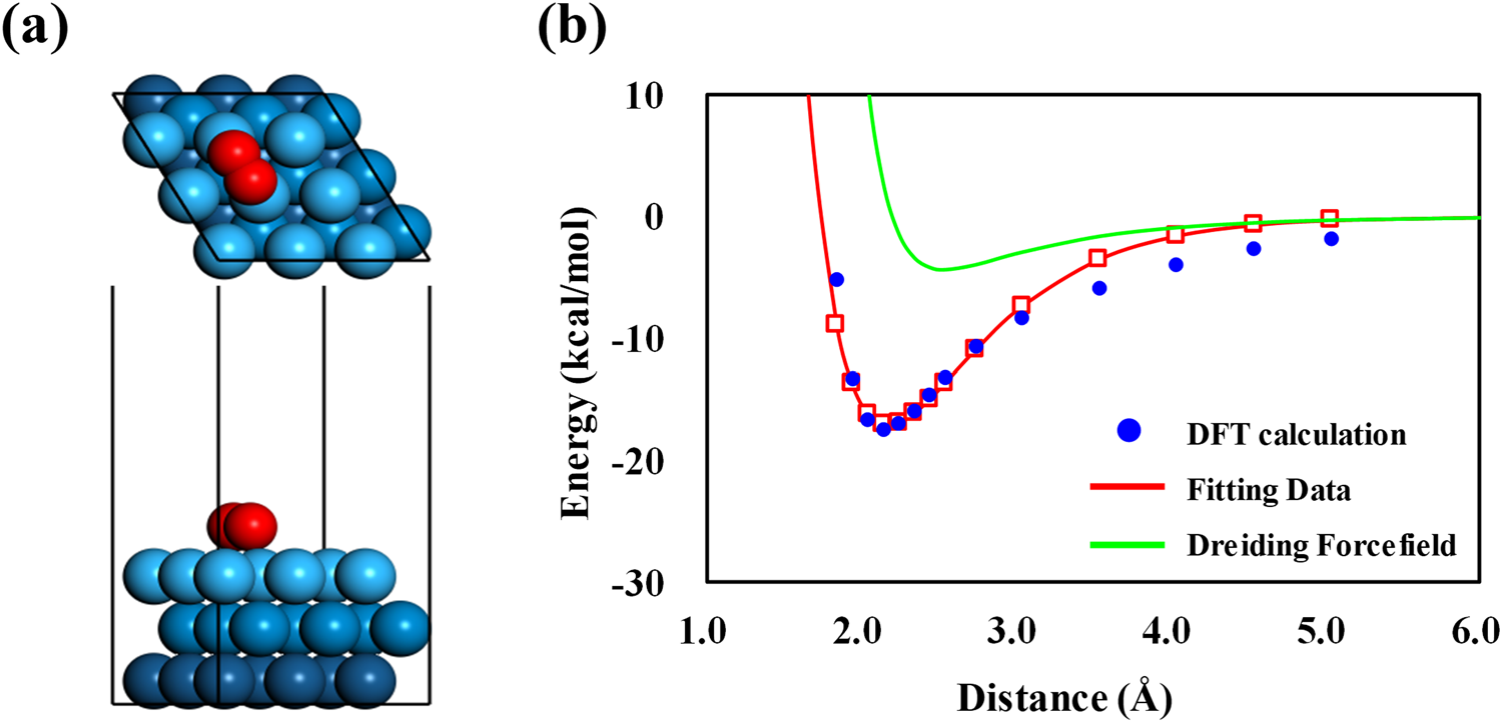
(**a**) The optimized structures of O_2_ molecules on the Pt (111) surface. (**b**) The change in potential energies according to changes in distance between O_2_ and the Pt (111) surface, as determined using DFT calculations, fitting data, and DREIDING force field.

We used the nonbonded interaction energies reported by Brunello et al.^42^ to describe the interactions of Pt atoms with Nafion, Aquivion, water, and hydronium ions. In addition, for the interaction energies between O_2_ and a Pt slab, we calculated van der Waals parameters via DFT calculations to describe detailed intermolecular interactions using a Pt (111) slab with three atomic layers with periodic boundary conditions (PBCs) of 8.324 × 8.324 × 25.000 Å^3^, as shown in Fig. 2. The DFT calculation details were the same as those used in the charge analysis (Sect. 2.2.1), and a semi-empirical dispersion correction (DFT-D) with the Tkatchenko–Scheffler scheme was additionally applied^43^. Band-structure calculations with *k*-points were performed with a 4 × 4 × 1 Monkhorst–Pack *k*-point mesh^44^.

### Model construction

To generate the Pt (111) surface and hydrated PFSA ionomers, we constructed a Pt (111) slab with five atomic layers with PBCs of 38.446 × 38.845 × 300.000 Å^3^. A *z*-direction length of 300 Å was used to prevent interaction beyond the PBCs. To construct hydrated PFSA ionomers on a Pt (111) surface, the *λ* (water molecules per sulfonic acid group) values were 2.92, 6.15, 9.77, and 13.83 for hydrated Nafion and Aquivion ionomers. The PFSA ionomers were composed of six polymer chains with 60 hydronium ions to maintain electrical neutrality. The Monte Carlo simulation in the Amorphous Cell module of the Materials Studio software^25^ was used to construct the initial configuration of hydrated PFSA ionomers on a Pt (111) surface.

### MD simulations

After the initial hydrated PFSA with Pt (111) surface models were constructed, the temperature was gradually increased to the operating temperature (353.15 K) of PEMFCs with canonical ensemble (NVT) for 1 ns. Consequently, 15 ns MD simulations were performed by NVT simulation to obtain the equilibrated structures. To simulate the permeation of O_2_ into PFSA ionomers, 735 O_2_ molecules with a thickness of 100 Å were placed on the equilibrated PFSA ionomers. After the O_2_ molecules were added, a total of 150 ns of NVT simulations at 353.15 K was performed. The last 10 ns of NVT simulation was used for data collection of the O_2_ permeation properties.

## Results and discussion

### van der Waals parameters for O_2_ and Pt

For a better description of the O_2_ permeation process inside a hydrated Nafion ionomer thin film on a Pt (111) surface, the atomic interaction curve between O_2_ and the Pt surface was reproduced by DFT under the framework of the DREIDING force field. Figure 2a shows O_2_ adsorbed onto the Pt surface, which was built for calculation of the adsorption energy as a function of the *z*-distance. The calculated adsorption energy as a function of distance was fitted to the Lennard–Jones potential in Fig. 2b, which well reproduced the results of DFT calculations. The Lennard–Jones potential function is shown in Eq. (2):

2 $$E=\varepsilon \left[{(\frac{{r}_{\mathrm{m}}}{r})}^{12}-2{(\frac{{r}_{\mathrm{m}}}{r})}^{6}\right]$$

where $E$ indicates the potential energy with changing distance $r$ and $\varepsilon$ is the depth of the potential well at distance ${r}_{\mathrm{m}}$. The values of $\varepsilon$ and ${r}_{\mathrm{m}}$ for the oxygen atoms in O_2_ molecules on a Pt surface are 4.070 kcal/mol and 2.338 Å, respectively. The fitted interaction well describes detailed interactions that the DREIDING force field cannot describe. Thus, the fitted interaction between O_2_ and Pt surfaces was used in the MD simulation to analyze the process of O_2_ permeation into a Nafion ionomer coated onto a Pt surface.

### Equilibrated structure

**Figure 3:**
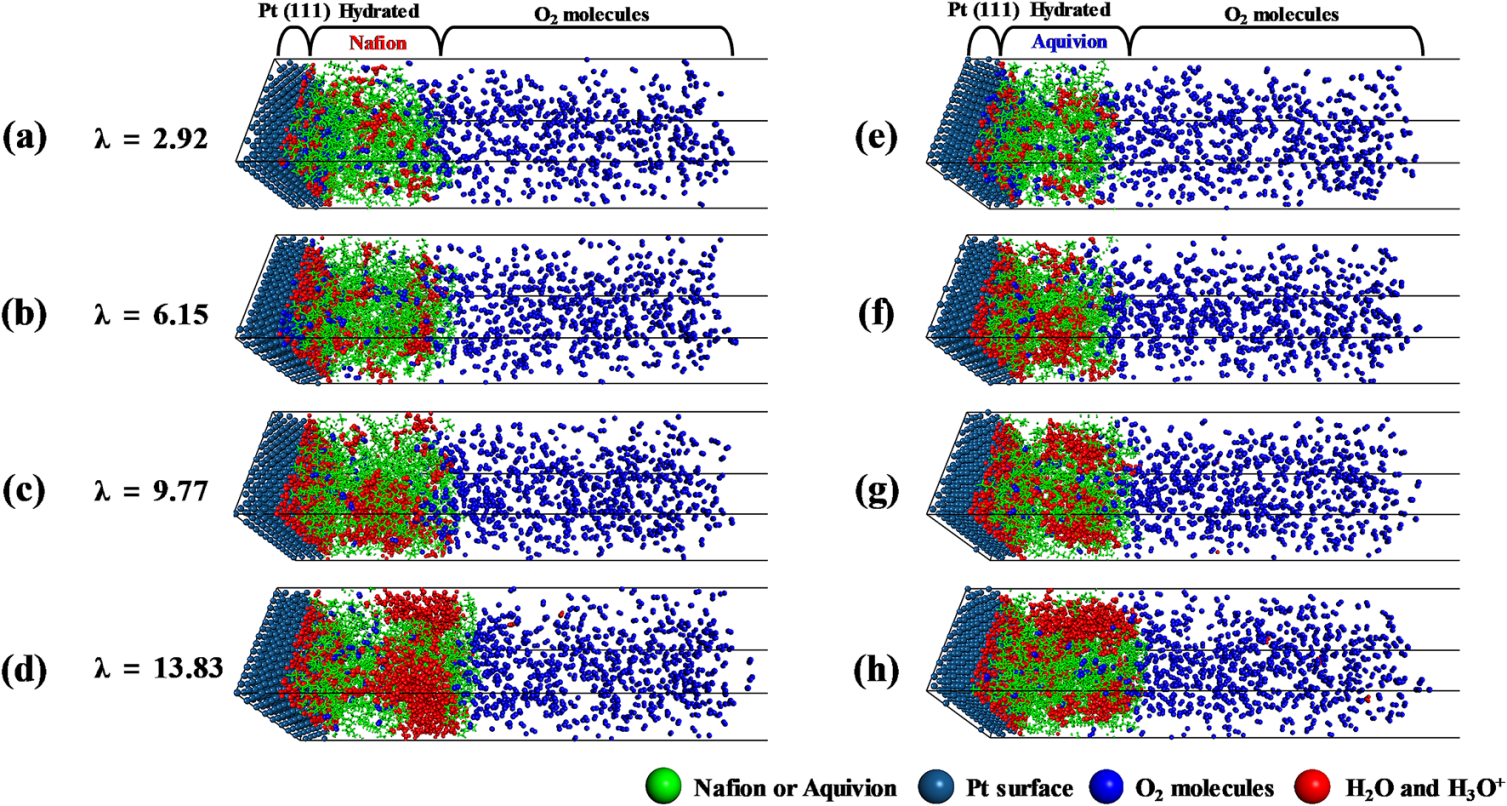
Snapshots of equilibrated systems comprising the Pt (111) surface, hydrated Nafion ionomers, and O_2_ molecules with *λ* values of (**a**) 2.92, (**b**) 6.15, (**c**) 9.77, and (**d**) 13.83; snapshots of equilibrated systems comprising the Pt (111) surface, hydrated Aquivion ionomers, and O_2_ molecules with *λ* values of (**e**) 2.92, (**f**) 6.15, (**g**) 9.77, and (**h**) 13.83.

Equilibrated structures in Fig. 3a–h were obtained from the data corresponding to the final 5 ns of the MD trajectories. Figure 3a–d show a hydrated Nafion thin film on a Pt surface, and Fig. 3e–h show a hydrated Aquivion thin film on a Pt surface, both under different hydration levels, *λ*. As *λ* increases, the Nafion and Aquivion films become gradually segregated into hydrophilic water clusters and hydrophobic regions with PTFE backbones of Nafion and Aquivion ionomer, respectively. Water molecules are predominantly adsorbed as a thin layer onto the Pt surface because they strongly interact with this surface^30^.

**Figure 4:**
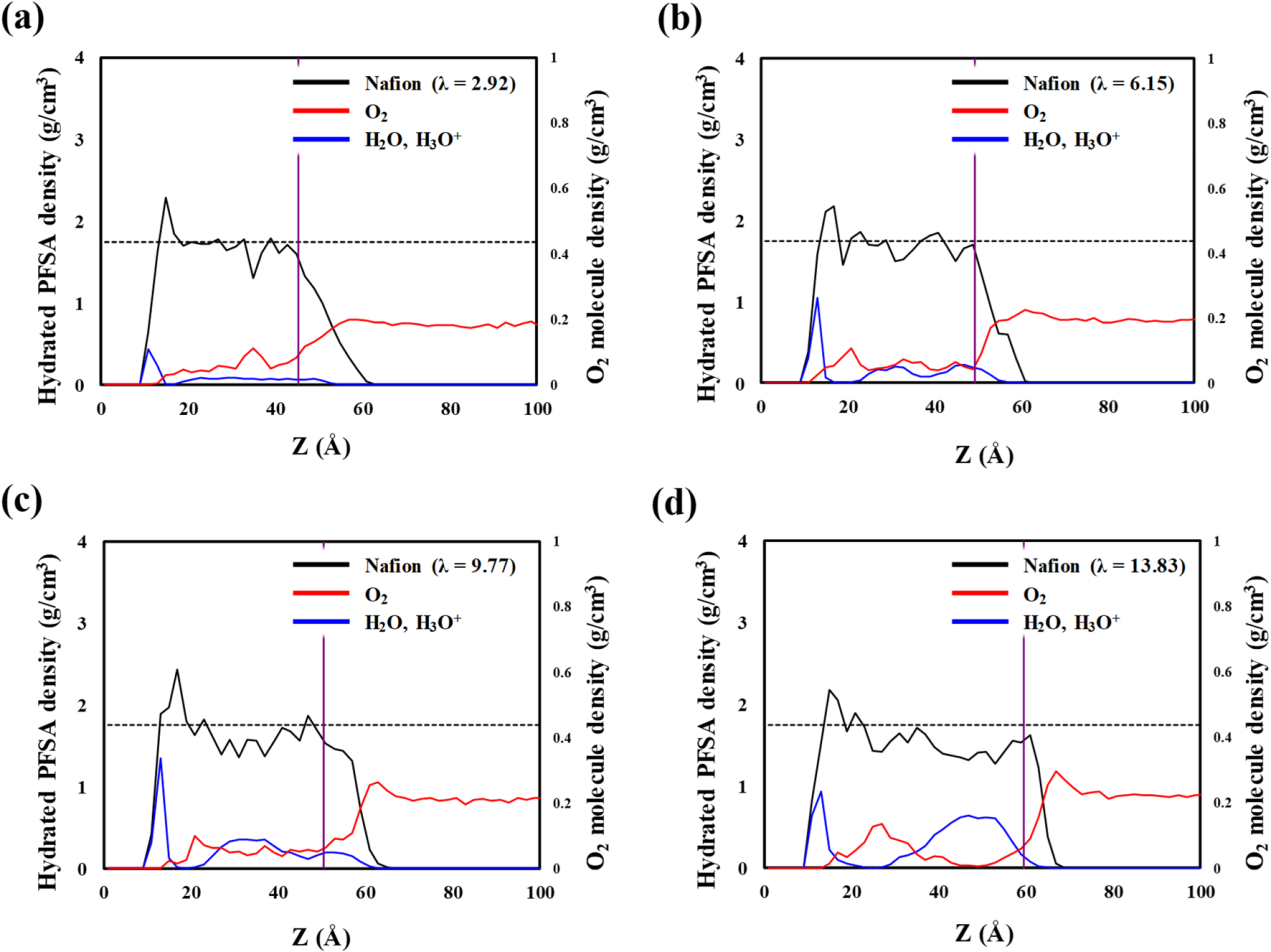
Density distributions of hydrated Nafion ionomer (with included water and hydronium ions; black lines), water and hydronium ions (blue lines), and O_2_ molecules (red lines) on the Pt (111) surface along the thickness direction, with *λ* values of (**a**) 2.92, (**b**) 6.15, (**c**) 9.77, and (**d**) 13.83. Dashed lines show the previously reported density for hydrated bulk Nafion^20^. The purple line indicates the point of O_2_ solvation occurs from the interface of gas/hydrated Nafion.

To quantitatively analyze the permeation of O_2_ molecules, we investigated the density profile of hydrated Nafion, water molecules with hydronium ions, and O_2_ molecules, as shown in Fig. 4a–d. Dotted lines represent the average density of a bulk Nafion membrane, as reported in our previous study^20^, which indicates that the density of hydrated Nafion on a Pt surface is not substantially different from that of a bulk membrane except for the density at the Nafion–Pt interface. As shown in the equilibrated structures in Fig. 3a–h, hydrated Nafion ionomers and water molecules with hydronium ions exhibit the highest density at the hydrated Nafion–Pt interface because of their strong attractive interaction. The purple line indicates the point of O_2_ solvation from the hydrated Nafion–gas interface, which is determined by the distance corresponding to the average number of total solvated O_2_ molecules. At this point, the density of O_2_ molecules abruptly decreases from its maximum value. These results are in good agreement with those of Jinnouchi et al.^24^, who reported that an energy barrier at the Nafion–gas interface dominates the solubility of O_2_ in hydrated Nafion. Inside hydrated Nafion, dissolved O_2_ molecules exhibit the highest density and water molecules with hydronium ions exhibit the lowest density. This trend becomes more discernible as the hydration level increases. These results suggest that O_2_ molecules are not preferentially positioned inside hydrophilic domains but rather at the interfacial regions between hydrophobic and hydrophilic regions.

**Figure 5:**
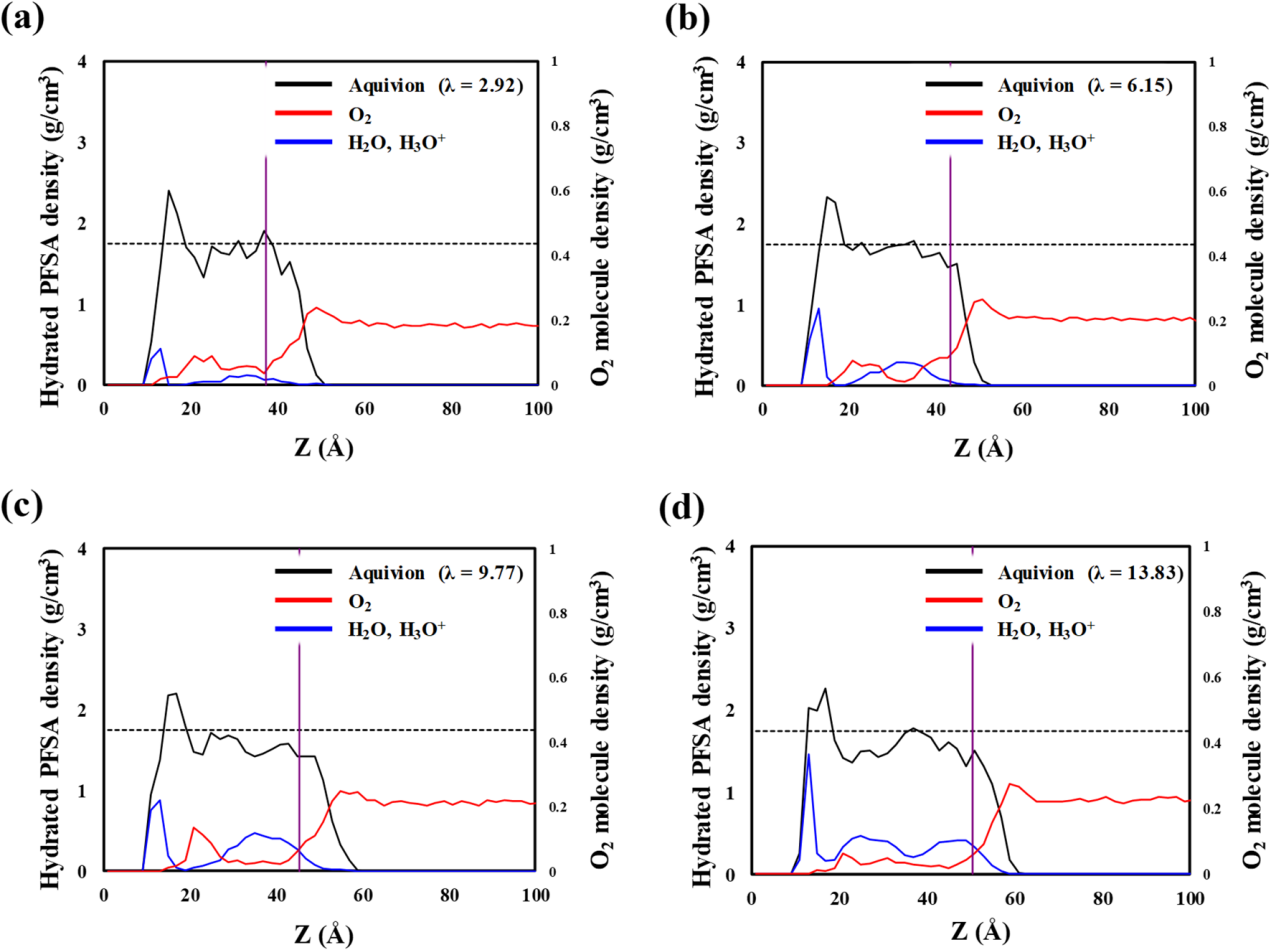
Density distributions of hydrated Aquivion ionomer (with included water and hydronium ions; black lines), water and hydronium ions (blue lines), and O_2_ molecules (red lines) on the Pt (111) surface along the thickness direction, with *λ* values of (**a**) 2.92, (**b**) 6.15, (**c**) 9.77, and (**d**) 13.83. Dashed lines show the previously reported density for hydrated bulk Aquivion^20^. The purple line indicates the point of O_2_ solvation occurs from the interface of gas/hydrated Nafion.

We also investigated the density profile of water molecules, hydronium ions, and O_2_ molecules in hydrated Aquivion; the results are shown in Fig. 5a–d. Like Nafion, hydrated Aquivion and water molecules with hydronium ions show the highest density at the Aquivion–Pt interface because of their strong attractive interaction. The hydrated Aquivion is thinner than the hydrated Nafion because of Aquivion’s shorter side chains and lower EW. The density of O_2_ molecules also abruptly decreases the maximum value at the distance indicated by the purple line, which represents the distance at which O_2_ solvation begins. Inside hydrated Aquivion, the dissolved O_2_ molecules exhibit the highest density and water molecules with hydronium ions exhibit the lowest density.

### O_2_ solubility and permeation

**Figure 6:**
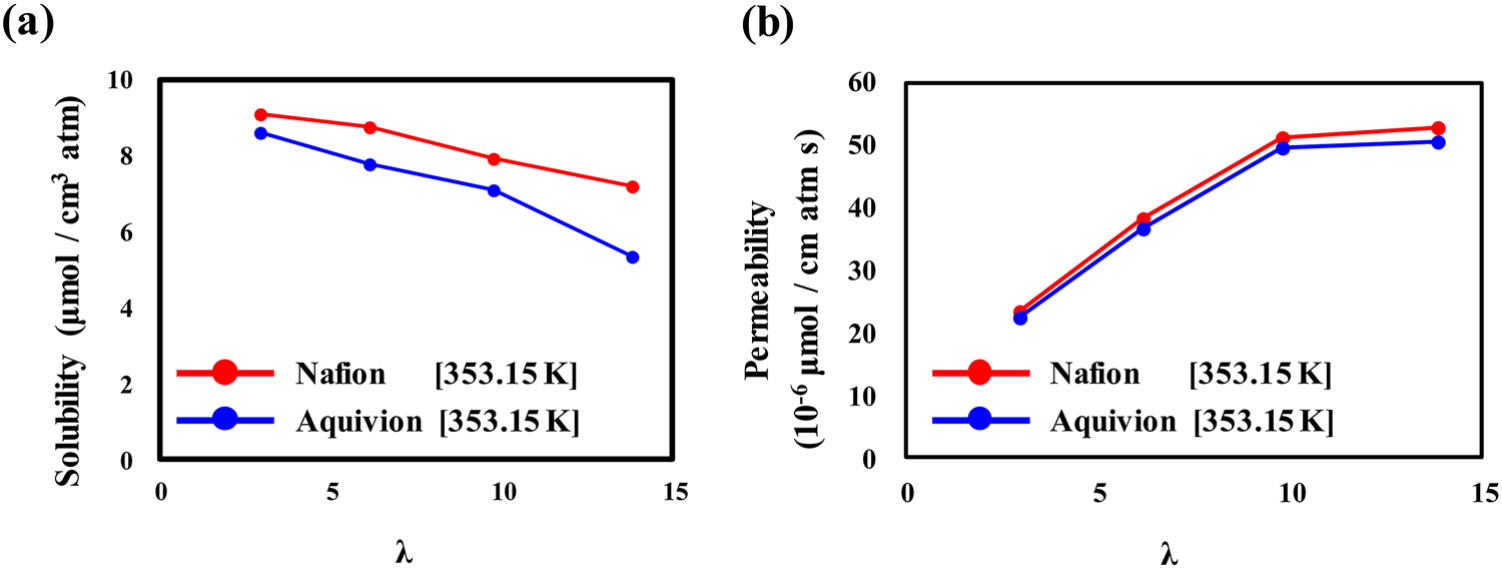
O_2_ (**a**) solubility and (**b**) permeability in hydrated Nafion and Aquivion on a Pt (111) surface.

To quantify the solvation of O_2_ molecules by hydration level, we calculated the solubility on the basis of the average number of dissolved O_2_ molecules at the distance indicated by the purple vertical line in Figs. 4 and 5. The solubility of Nafion and Aquivion ionomers as a function of the hydration level is presented in Fig. 6a. The solubility of O_2_ decreases with increasing hydration level in both the Nafion and Aquivion ionomers. Because the interfacial region between hydrophilic and hydrophobic domains becomes relatively limited when the net ionomer volume increases under hydration, the number of sites available for dissolved O_2_ molecules also becomes limited. Therefore, the ionomers exhibit low O_2_ solubility at high hydration levels (*λ*). At the same *λ*, the O_2_ solubility is higher in the Nafion ionomer than in the Aquivion ionomer, which means that the solvation of O_2_ molecules in the Aquivion ionomer is restricted compared with that in the Nafion ionomer.

Using the solubility values, we derived the permeability coefficient of O_2_ molecules. Gases permeate through ionomers via a solution-diffusion mechanism, where the dissolved gas molecules diffuse into the ionomers^45^. Thus, the permeability coefficient *P* is described by the equation

3 $$P=D\times S$$

where *D* is the diffusion coefficient and *S* is solubility. The diffusion coefficient in our system was obtained from the self-diffusion coefficient of O_2_ molecules in the bulk structures of Nafion and Aquivion. The calculated permeability coefficients for O_2_ molecules in Nafion and Aquivion ionomer are shown in Fig. 6b; these values are consistent with those reported by Baschetti et al.^15^. With increasing hydration level *λ*, the permeability of O_2_ in both Nafion and Aquivion ionomers increases until *λ* = 9.77. At *λ* > 9.77, the permeability increases slightly because, despite the diminished solubility, the self-diffusion coefficient of O_2_ in a bulk ionomer membrane increases with increasing hydration level; thus, permeability increases with increasing hydration level. In our previous work^20^, we reported that O_2_ molecules exhibit greater diffusion in Aquivion ionomers than in Nafion ionomers because of the Aquivion ionomers’ better-developed water channels. However, at the same hydration level *λ*, both the permeability and solubility of O_2_ are higher in Nafion ionomer than in Aquivion ionomer. This result means that solubility is more critical to permeability, which is dominated by the availability of solvation sites for dissolved O_2_ molecules in the ionomer.

### RDF analysis

**Figure 7:**
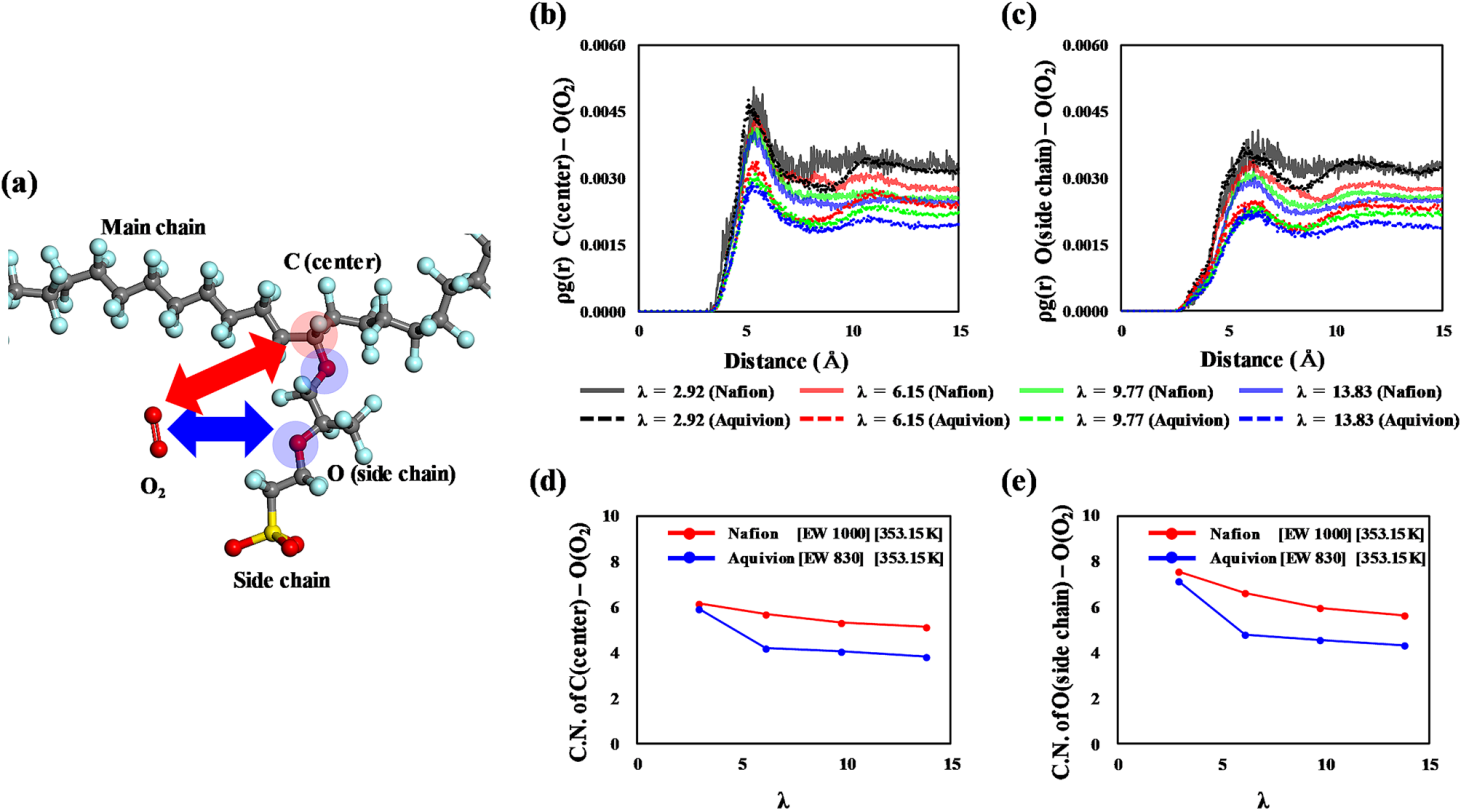
(**a**) Molecular model with the atomic definition for O_2_ location analysis using RDFs. RDFs at (**b**) C(center)–O(O_2_) and **c**) O(side chain)–O(O_2_) and the first coordination number of (**d**) C(center)–O(O_2_) and (**e**) O(side chain)–O(O_2_).

The interface region between hydrophilic and hydrophobic domains in a hydrated PFSA ionomer, where O_2_ molecules preferentially dissolve, is most likely to be the side-chain part above the sulfonic acid groups. That is, O_2_ solvation mainly occurs at the side chains of the ionomers. Thus, the difference in solubility between Nafion and Aquivion is reasonably deduced to arise from their different side-chain structures. In this regard, we analyzed the structure between O_2_ and the main component of the Nafion and Aquivion side-chain structures to understand the difference in O_2_ solubility between them. As shown in Fig. 7a, we analyzed correlations between Carbon(center)–Oxygen(O_2_) and Oxygen(side chain)–Oxygen(O_2_) using RDFs. The RDF of each pair is described by the following equation:

4 $$g_{A - B} \left( r \right) = \left( {\frac{{n_{B} }}{{4\pi r^{2} dr}}} \right)/\left( {\frac{{N_{B} }}{V}} \right)$$

where ${n}_{B}$ is the number of *B* particles located at a distance *r* in a shell of thickness $dr$ from particle *A*, *N*_*B*_ is the number of *B* particles in the system, and *V* is the total volume of the system; *N*_*B*_/*V* can be represented by the number density, *ρ*. The *ρg*(*r*) of each pair of Nafion and Aquivion ionomers is presented in Fig. 7b, c, respectively. The *ρg*(*r*) of both C(center)–O(O_2_) and O(side chain)–O(O_2_) shows the first peak at 7.87 Å and 8.51 Å, respectively. These values decrease with increasing hydration level. At the same hydration level, the *ρg*(*r*) of both C(center)–O(O_2_) and O(side chain)–O(O_2_) is greater in the Nafion ionomer than in the Aquivion ionomer. This result means that O_2_ has a more favorable correlation with C(center) and O(side chain) in the Nafion ionomer than with those in the Aquivion ionomer. The first coordination number (CN) at the distance of the first peak was calculated (Table 2). As shown in Fig. 7d, e, the CN of C(center)–O(O_2_) and O(side chain)–O(O_2_) pairs also decreases as the hydration level increases with increasing *ρg*(*r*). At the same hydration level, the Nafion ionomer shows a higher CN of each pair than the Aquivion ionomer. This result suggests that more O_2_ molecules are coordinated to the side chain of the Nafion ionomer than to that of the Aquivion ionomer, especially to the side chains’ oxygen atoms, which results in greater solubility of O_2_ in the Nafion ionomer. In addition, we note that Aquivion shows a similar CN as Nafion at *λ* = 2.92. As described in the previous section, we also observed that the difference in solubility and permeability is more discernible at a hydration level greater than *λ* = 2.92. At a low hydration level, the site for O_2_ solvation is not significantly different due to only a few water molecules located near the side chain leading to a lack of phase segregation, and O_2_ molecules can be easier located near the side chain of PFSA than other water contents. However, as the hydration level increases, the solvation sites of O_2_ molecules become limited to the side-chain region of the ionomer due to the water molecules formed the water cluster near sulfonic acid groups of PFSA. Although the water molecules formed the water cluster near sulfonic acid groups, O_2_ molecules can be located near the side chain of Nafion easier than Aquivion because the side chain of Nafion is longer than that of Aquivion, leading to higher CNs of Nafion. The CN of C(center)–O(O_2_) and O(side chain)–O(O_2_) pairs of Aquivion were sharply decreased at λ = 6.15 because we assume that the water cluster can be formed in Aquivion easier than Nafion due to higher sulfonic acid group concentration. After that, the CNs of Aquivion at 9.77 to 13.83 was gradually decreased with developing water clusters and channels near the side chain. Consequently, Nafion and Aquivion exhibit an observable difference in solubility at higher hydration levels.

**Table 2:** The coordination numbers of Nafion and Aquivion on a Pt surface at 353.15 K, where the water contents (*λ*) were varied.

Pairs	Ionomer	*λ* = 2.92	*λ* = 6.15	*λ* = 9.77	*λ* = 13.83
C(center)–O(O_2_) (7.87 Å)	Nafion	6.18	5.68	5.35	5.15
	Aquivion	5.91	4.22	4.05	3.82
O(side chain)–O(O_2_) (8.51 Å)	Nafion	7.56	6.60	5.94	5.64
	Aquivion	7.14	4.79	4.55	4.30

## Conclusion

We prepared two types of hydrated PFSA ionomers on a Pt (111) surface and varied their water contents (2.92 ≤ *λ* ≤ 13.83) to analyze the distribution morphologies and permeability properties of O_2_ molecules in detail using full atomistic MD simulations at the operating temperature of PEMFCs (353.15 K). The hydrated PFSA ionomer structures were prepared using Nafion (long side chain) and Aquivion (short side chain). In the MD simulations, O_2_ molecules were gradually permeated into hydrated PFSA ionomers on the Pt surface. Density profile analysis for hydrated PFSA ionomers was performed to calculate the O_2_ solubility in hydrated PFSA ionomers with various *λ*. The O_2_ solubility in hydrated Nafion ionomers on the Pt surface was higher than that in hydrated Aquivion ionomers on the Pt surface at the same *λ* values. The RDF analysis showed that the first CN of C(center)–O(O_2_) and O(side chain)–O(O_2_) pairs of hydrated Nafion ionomers was greater than that of hydrated Aquivion ionomers at the same water content. These results suggest that more O_2_ molecules are coordinated to the side chains of the Nafion ionomer than to those of the Aquivion ionomer, especially oxygen atoms, which results in greater O_2_ solubility in the Nafion ionomer.
